# Radiology’s role in providing and ensuring access to reproductive care

**DOI:** 10.1093/bjr/tqaf071

**Published:** 2025-03-31

**Authors:** Katherine Frederick-Dyer, Mindy M Horrow, Theresa M Caridi, Rochelle F Andreotti

**Affiliations:** Department of Radiology and Radiological Sciences, Vanderbilt University Medical Center, Nashville, TN 37232-2675, United States; Department of Radiology, Jefferson/Einstein Healthcare, Philadelphia, PA 19141, United States; Department of Radiology, University of Alabama at Birmingham, Birmingham, AL 35294, United States; Department of Radiology and Radiological Sciences, Vanderbilt University College of Medicine, Nashville, TN, 37232-2675, United States; Department of Obstetrics and Gynecology, Vanderbilt University College of Medicine, Nashville, TN, 37232-2675, United States

**Keywords:** abortion, Dobbs decision, reproductive healthcare, United States

## Abstract

The US Supreme Court Dobbs decision has significantly impacted reproductive healthcare in the United States. While obstetricians are most directly affected, radiologists, radiation oncologists, and medical imaging professionals are also involved in the care of pregnant patients, and these legislative changes can alter our practices. In this commentary, we describe examples of how this reproductive legal landscape has changed the practice of interventional radiology in states affected by abortion bans, discuss some of the radiologist lead advocacy efforts for the preservation of reproductive healthcare, and review the Society of Radiologists in Ultrasound’s first trimester ultrasound consensus statement clarifying and standardizing medical terminology frequently used in radiologist reports. The radiology community has a responsibility to advocate for the preservation of reproductive health and the patient–physician relationship.

## Introduction

On June 24, 2022, the Supreme Court of the United States (SCOTUS) decided Dobbs v. Jackson Women’s Health Organization, overturning Roe v. Wade and ending federal protections for the constitutional right to abortion prior to foetal viability. This ruling undid 50 years of legal precedent, concluding that the “Constitution does not confer a right to abortion”.^1^ Instead, the issue is left to the discretion of state governments, giving them the ability to restrict or outlaw abortion entirely. Twenty-one states now ban abortion or restrict the procedure to a gestational age earlier than foetal viability. Thirteen states have enacted almost complete abortion bans, with very limited exceptions generally including pregnancies endangering the life of the mother, ectopic pregnancy, and molar pregnancy.^2^ Many of these bans do not include exceptions for rape, incest, or fatal foetal anomalies,^3^ and physicians who perform or prescribe medication for illegal abortions in these states will face felony charges resulting in loss of medical licensure and penalties including significant prison time and fines.^4–6^

Obstetricians, who primarily navigate this variable legislative interference in the patient–physician relationship, are faced with the unreasonable task of determining when their patients are sick enough such that they can proceed with evidence-based medical care without risking a career ending felony charge. The ramifications to women’s healthcare are becoming apparent. Trends in Texas may be informative since the Texas State Legislature banned abortion at 5 weeks gestation almost 1 year prior to the Supreme Court decision in 2022. According to an analysis of publicly available data from the US Centers for Disease Control and Prevention, there was a 56% increase in maternal mortality in Texas from 2019 to 2022 compared to 11% nationwide.^7^

Radiologists share responsibility as providers of both diagnostic and interventional care for pregnant patients. In the therapeutic realm, interventional radiologists and radiation oncologists may face some of the same difficult decisions that obstetricians now routinely encounter. In this essay, we will explore some of the ways that interventional radiologists in the United States have been affected by the changing post-Dobbs legal climate and how the radiology community has contributed to the preservation of evidence-based obstetrical care through both advocacy and multidisciplinary standardization of the interpretive lexicon.

## Impact of the supreme court decision on reproductive health practices in interventional radiology

The SCOTUS decision on reproductive rights has significant implications for interventional radiologists (IRs), affecting both direct reproductive health therapies and the broader healthcare context in which IRs operate. While IRs face numerous headwinds navigating current reproductive health therapies, we will focus on 3 to illustrate challenges in IR practice, with significant variability depending on the interventionalist’s location: (1) uterine artery embolization (UAE) for ectopic pregnancy, (2) hysterosalpingography (HSG) and fallopian tube recanalization (FTR), and (3) training future IRs.

**Figure 1.**
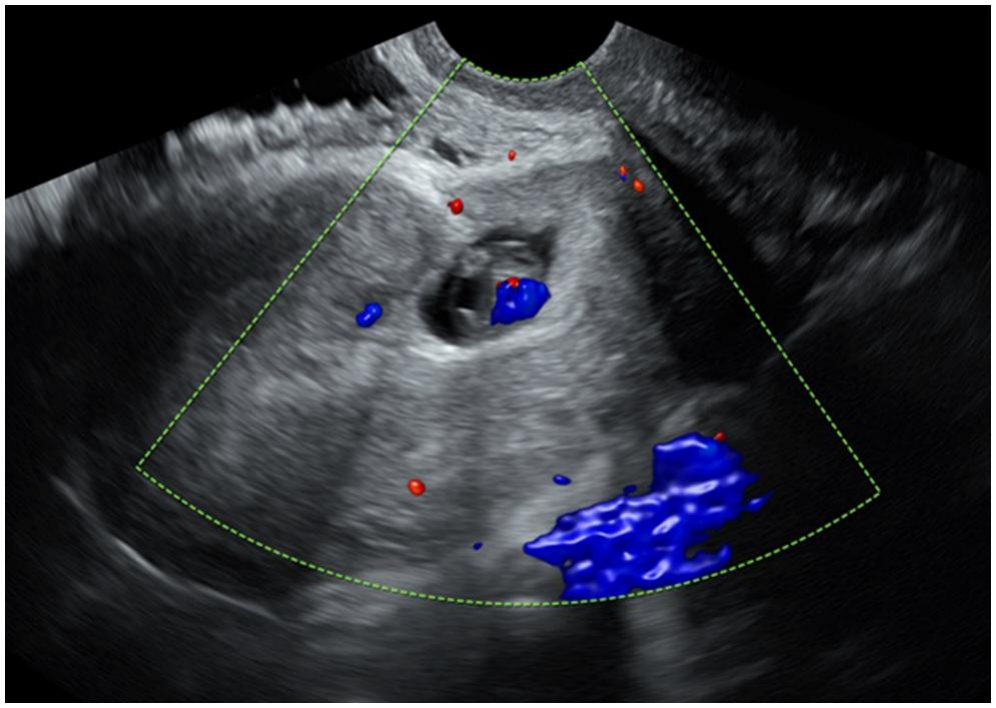
A sagittal transvaginal ultrasound image with colour Doppler shows a gestational sac implanted in the caesarean scar site, separate from the endometrium. Cardiac activity is present in the 7-week embryo. Despite the intrauterine implantation of this pregnancy, it is considered an ectopic pregnancy.

UAE is a minimally invasive procedure used for a variety of pelvic conditions including treatment of ectopic pregnancies, particularly when the pregnancy is implanted at the cervix or the interstitial part of the fallopian tube.^8^ This procedure involves the selective occlusion of the uterine arteries to reduce blood flow to the ectopic pregnancy thereby causing it to regress, with the benefits of potentially preserving fertility in properly selected patients by avoiding salpingectomy. However, this effectively and intentionally terminates a gestation, and results in confusion and hesitancy to provide this service in states with abortion bans that could result in felony charges. This becomes especially problematic in cases of caesarean scar ectopic pregnancy, which are extremely risky to the life of the mother but can occasionally be carried to viability^9^ (Figure 1).

**Figure 2.**
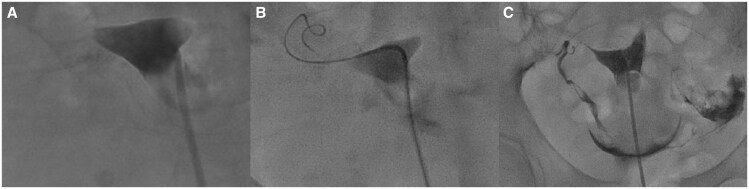
(A) HSG demonstrating occluded fallopian tubes. (B) FTR intraprocedural image showing wire recanalization of the right fallopian tube. (C) Post-FTR HSG image demonstrating patency of both fallopian tubes.

The radiology procedures HSG and FTR highlight the changes in referrals due to the broader reproductive health care climate. HSG is performed to assess fallopian tube patency and for other uterine/tubal abnormalities. In cases of tubal blockage patients can be referred to IR for FTR, where fallopian tubes are opened either through irrigation or with a wire/catheter technique^10^ (Figure 2). The use of HSG and FTR is typically predicated on the presence of a Reproductive Endocrinology and Infertility (REI) division of Gynecology. In states where reproductive rights are continuously challenged, REI providers have been restricted in their ability to fully support their patients’ pregnancy and pregnancy loss journeys, choosing to relocate or forgo this aspect of gynaecologic practice, thus impacting the practice patterns of HSGs and FTRs. For example, the Alabama State Supreme Court ruled in February 2024 that fertilized eggs and embryos have the same status as children as a direct consequence of the state’s abortion ban. As a result, many of the state’s reproductive healthcare centres temporarily paused in vitro fertilization (IVF) practices due to fear of legal consequences related to the potential disposal of non-implanted embryos.^11^ Equating embryos to children puts these REI practices in question and thereby impacts referrals to IR practices.

Finally, impacts on current reproductive health practices and the broader healthcare landscape also affect future generations of IRs. Current trends in obstetrical training may be informative. While obstetrical residency slots across the nation still fill, there is a decrease in the number of residency applications to programmes in states with abortion bans.^12^ Furthermore, obstetrical residents who train in states with abortion bans must travel to obtain training in abortion care.^13^ While these trends do not seem to affect interventional radiology residency programmes specifically, IR residents who train in states with abortion bans will not have opportunity to develop the skills to offer full spectrum IR reproductive health services.

## Radiologist advocacy in the post-Dobbs era

While obstetricians have been the primary advocates for pregnant patients in the post-Dobbs era, radiologists and other medical imaging professionals are realizing the importance of adding their voices as physicians caring for pregnant patients and consultants supporting their obstetrical colleagues.

Shortly after Dobbs was decided, several major radiology societies^14–16^ issued statements speaking out against interference in the patient-physician relationship, including the ACR (American College of Radiology), the RSNA (Radiological Society of North America), and the SIR (Society of Interventional Radiologists). The RSNA statement specifically expressed disappointment in the SCOTUS decision to overturn Roe vs Wade, noting that the decision “endangers women’s lives and exacerbates health can inequities.”^15^ The SIR deemed the decision a “serious threat to the physician-patient relationship and physicians’ ability to deliver safe and effective care to our patients during the most critical moments of their lives” and declared that “Physicians and patients should not have to worry that the decision being made bedside is in any way influenced by consideration other than the wellbeing of the patient.”^16^ As the major politically centric radiology organization in the United States, the ACR position was that the patient-physician relationship is “sacred” and “must not be jeopardized by non-medical outside interference, including federal, state and local government intrusions beyond public health measures.”^14^

**Figure 3.**
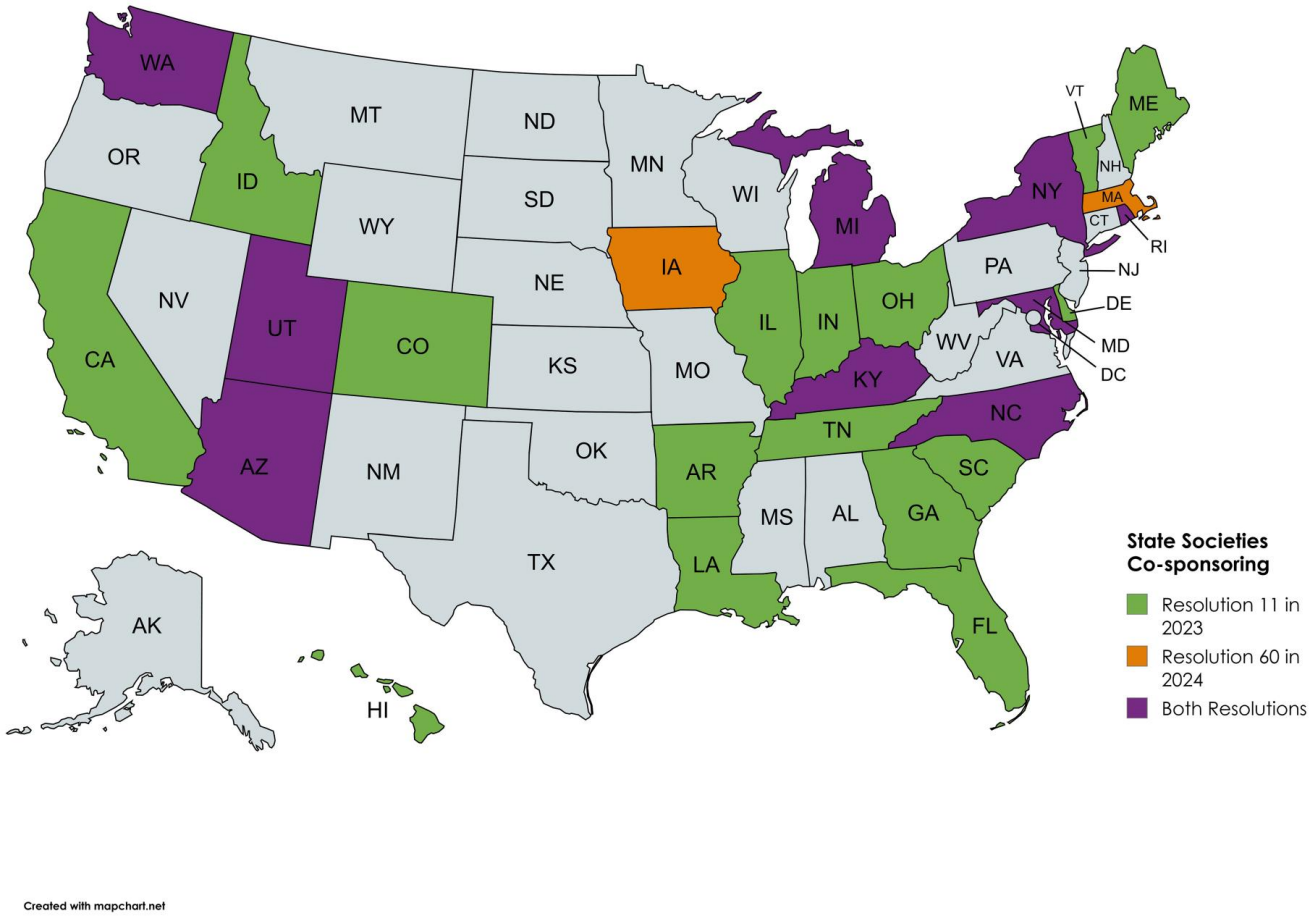
Map of American College of Radiology State Chapters Co-Sponsoring Resolution 11 “ACR Opposes Interference in the Physician-Patient Relationship” in 2023 and Resolution 60 “Resolution Supporting Abortion as an Essential Component of Health Care” in 2024.

At a grassroots level, over 180 radiologists and medical physicists from institutions and practices across the country signed a petition supporting reproductive rights, stating that the signers “staunchly support patient safety and autonomy, in opposition to the SCOTUS decision to overturn Roe v Wade.”^17^ This groundswell effort resulted in the ACR’s adoption of Resolution 11 in 2023, stating that “the ACR affirms that government and other third-party interference in evidence-based medical care may compromise the sanctity of the patient-physician relationship and undermine the provision of quality health care” and asked that the society “oppose any government regulation or legislative action that would criminalize radiologists, interventional radiologists, nuclear medicine physicians, radiation oncologists, physicists, and other medical imaging professionals for providing evidence-based medical care within the scope of their training, professional judgement, and nationally recognized professional practice guidelines.”^18^ The Resolution, titled “ACR Opposes Interference in the Physician-Patient Relationship,” was co-sponsored by 25 state chapters from across the country (Figure 3) as well as the Council Steering Committee and Council of Affiliated Regional Radiation Oncology Societies and was unanimously adopted by the Council.

During the summer of 2023, the American Association of Women in Radiology (AAWR) formed an Advocacy Committee which included a Reproductive Rights subgroup. This subgroup successfully campaigned for the ACR adoption of Resolution 60 in 2024, supporting abortion as an essential component of health care and as an evidence-based medical care option thereby joining more than 75 healthcare organizations with this affirmation. The resolution was co-sponsored by several groups including the ACR Resident and Fellow Sections, the Young Physicians Section, Council of Affiliated Regional Radiation Oncology Societies, and 11 state chapters (Figure 3).^19^

Members of the AAWR Reproductive Rights Advocacy Subgroup also advocated for pregnant patients by promoting best practices of imaging pregnant or potentially pregnant patients with ionizing radiation as detailed in the ACR-SPR (Society for Pediatric Radiology) Practice Parameters.^20^ The authors caution radiologists practicing in states with abortion bans that there may be motivation for patients to refuse pregnancy testing prior to an exam or even conceal the possibility of being pregnant. They emphasize respect for the patient’s privacy and autonomy but suggest (1) that information regarding potential risk of radiation exposure to a pregnancy be easily accessible to all patients, (2) that societies continue to provide and update guidance regarding which procedures require pregnancy testing, and (3) societies offer advice on approaching informed consent discussions for pregnancy testing in patients with privacy concerns.^21^

## Society of radiologists in ultrasound multispecialty consensus on first trimester ultrasound

It is not an overstatement that diagnostic ultrasound has revolutionized reproductive care of women. The earliest medical publication on diagnostic ultrasound in 1958 included images of ovarian cysts, fibroids, and pregnancies at 14 and 34 weeks with polyhydramnios and twins.^22^ Routine obstetric ultrasound imaging began in the 1960’s, allowing the first direct view of human foetal development and expanded dramatically in subsequent decades. As ultrasound technology evolved, so did its uses and with it an entire descriptive vocabulary and scientific literature to aid in interpretation. However, with great strides in pre-natal imaging also came the need for evidence-based consensus on issues with the potential to cause inadvertent harm in the first trimester such as the definitive diagnosis of miscarriage and ectopic pregnancy (EP). A landmark publication from the Society of Radiologists in Ultrasound (SRU) in 2013^23^ addressed the criteria for non-viability in the first trimester, standards which have stood the test of time with reproducibility in large population series.^24^

Prompted by the US Supreme Court Dobb’s decision, an SRU multispecialty panel convened in 2024 to address the terminology for first trimester imaging to promote the use of standard medically correct terminology that could be used safely in any state, as well as avoiding language that can be misconstrued by patients. This lexicon includes preferred terms, acceptable synonyms, and terms to avoid.^25^ The panel reviewed the scientific literature, accounted for the clinical and diagnostic needs of the participating medical specialties, and addressed patient preferences. Highlights are summarized in Tables 1 and 2.

**Table 1.** Definitions from SRU First Trimester Lexicon.

Terms	Definitions
Gestational Age	Duration of pregnancy, first trimester ≤13 weeks, 6 days
Cardiac activity	Rhythmic pulsations in embryo, replaces heartbeat
Intrauterine pregnancy/IUP	Normally located pregnancy
Ectopic pregnancy	Pregnancy implanted in abnormal location either extra or intra uterine (Figure 1)
Definitive IUP or EP	Requires a yolk sac or embryo
Probable IUP or EP	Empty gestational sac or tubal ring
Pregnancy of unknown location	No evidence of a definite or probable IUP or EP
Early pregnancy loss (EPL) Concerning Diagnostic In progress Incomplete Completed	Intrauterine pregnancy that will not progress, used with modifiers Normally located gestational sac (GS) with findings suggesting it may not progress Normally located GS with definitive findings that it will not progress GS in lower uterine cavity or endocervical canal in process of expulsion Residual tissue usually with vascularity after an EPL No persistent GS or tissue after an EPL

**Table 2.** Terms to avoid from SRU First Trimester Lexicon.

Terms	Reasons to avoid	Lexicon term
Viable, living, live	“Viability” is defined as ability to survive in extrauterine environment. “Live” is too personifying for the first trimester.	Cardiac activity
Heartbeat, heart motion	“Heart” implies a fully formed organ	Cardiac activity
Pregnancy failure	“Failure” may be misinterpreted by patients	Early pregnancy loss used with one of 5 descriptors (concerning, diagnostic, in progress, incomplete, completed)

As physicians who participate in the care of women during early pregnancy, radiologists must not only provide high-quality diagnostic ultrasound imaging with evidence-based interpretations, but must use a vocabulary that is clearly defined, scientifically based, and free of bias. Use of a standard lexicon is essential to providing quality care particularly at a time where the intersection of politics, religion, and medicine has become increasingly fragmented and contentious. This is the goal of the SRU first trimester lexicon.

## Conclusion

Given the recent United States 2024 election results, it is unlikely there will be a federal resolution to these significant reproductive healthcare issues in the near future. While a few state ballot measures were successful in protecting reproductive rights, many abortion bans remain in effect and will likely persist for the foreseeable future. Radiologists must continue to protect and care for our patients as best we can. We must carefully consider the wording of our reports in order to protect both patients and their caregivers, navigate legislation to provide optimal care, and continue to advocate for our patients and the sanctity of the patient-physician relationship.
